# Comparative Study between Direct and Pseudomorphic Transformation of Rice Husk Ash into MFI-Type Zeolite

**DOI:** 10.3390/molecules23010001

**Published:** 2017-12-21

**Authors:** Hallah Ahmad Alyosef, Hans Roggendorf, Denise Schneider, Alexandra Inayat, Julia Welscher, Wilhelm Schwieger, Tom Münster, Gert Kloess, Suzan Ibrahim, Dirk Enke

**Affiliations:** 1 Institute of Chemical Technology, Universität Leipzig, Linnéstr. 3, 04103 Leipzig, Germany; hallah.alyosef81@gmail.com (H.A.A.); denise.schneider@uni-leipzig.de (D.S.); 2 Institute of Physics, Martin-Luther-University Halle-Wittenberg, 06120 Halle (Saale), Germany; hans.roggendorf@physik.uni-halle.de; 3 Institute of Chemical Reaction Engineering, Friedrich-Alexander University Erlangen-Nürnberg (FAU), Egerlandstr. 3, 91058 Erlangen, Germany; alexandra.inayat@fau.de (A.I.); julia.welscher@fau.de (J.W.); wilhelm.schwieger@fau.de (W.S.); 4 Institute of Mineralogy, Crystallography and Materials Science, Universität Leipzig, Scharnhorststrasse 20, 04275 Leipzig, Germany; tom.muenster@gmx.de (T.M.); kloess@uni-leipzig.de (G.K.); 5 Central Metallurgical Research & Development Institute (CMRDI), P.O. Box 87, Helwan 11421, Egypt; suzansibrahim@gmail.com

**Keywords:** hydrothermal treatment, amorphous, rice husk ash, MCM-41, MFI-type zeolite, pseudomorphic transformation

## Abstract

Pre-shaped mesoporous amorphous rice husk ash (RHA) and MCM-41 derived from RHA as a silica source were transformed into MFI-type zeolites using two different structure-directing agents. Tetrapropylammonium hydroxide (TPAOH) was utilized as an alkali source for silica dissolution and structure control during the direct transformation of RHA into zeolite. A monopropylamine (PA)-containing alkaline solution (NaOH) was used for the pseudomorphic transformation of RHA or MCM-41 into zeolite. The hydrothermal conversion of RHA or MCM-41 into MFI-type zeolites was investigated as a function of reaction time at 175 °C. With PA as template, the crystallization took place inside and on the outer surface of RHA or MCM-41 without losing the original shape of the initial silica sources, while TPAOH led to the formation of conventional MFI-type zeolite crystals due to the complete dissolution of RHA. The final products were characterized by X-ray diffraction, nitrogen adsorption, scanning electron microscopy, and optical emission spectroscopy.

## 1. Introduction

Agricultural products and agro-residues attract widespread attention as biomasses for renewable energy generation. This is due to their high content of organic components, i.e., hemicellulose, lignin, and cellulose [1,2]. After burning, many agricultural crops, such as miscanthus, or their by-products, such as rice husk (RH), cereal remnant pellets, and wheat straw, leave an inorganic residue (ash) which is rich in silica. Thus, it is considered a potential raw material for the preparation of high-value silica products [1,2]. Several studies have introduced the concept of recovering silica from such biomass materials, and they concluded that the silica yield of rice husk ash (RHA) is the highest among the above-mentioned biomass products [1,2].

Nowadays, the disposal of RH has become a problem for the environment, since it generates excessive air pollution during its uncontrolled burning [3]. Nevertheless, its burning provides abundant and cheap alternatives for silica in many industrial applications, such as the preparation of elementary silicon [4]. Similarly to various commercial silica shapes, such as spherical LiChrospher 100, LiChrospher 60 [5], microporous high silica DAY cylinders (extrudates) [6], and porous glass monoliths [7,8], RHA has been considered as a promising material for the synthesis of hierarchically structured meso-/macroporous MCM-41 or MCM-48 silicas via pseudomorphic transformation [5,6,7,8,9]. This procedure is mainly based on the utilization of either hexadecyltrimethylammonium bromide (CTAB) as a mesopore template in the presence of sodium hydroxide (NaOH) [5] or hexadecyltrimethylammonium hydroxide (CTAOH). In the latter case, the dissolution and reorganization of silica during the transformation process is combined in one molecule [6,7,8,9].

In addition, commercial porous glass beads have been transformed into ZSM-5 zeolite with micro-/macroporous characteristics while retaining the original size and macroscopic morphology of the initial glass beads. During the hydrothermal transformation into ZSM-5 zeolite, dipropylamine (DPA) or monopropylamine (PA) have been adapted as structure-directing agents (SDA). The transformation was carried out under rotation at 175 °C for 72 h [10,11]. The obtained systems with a bimodal pore structure (micro/macro, meso/macro) might be applied in separation and catalysis [11,12,13,14].

The use of different pre-shaped biogenic silica species, such as diatomite or RHA, as an amorphous silica precursor for the preparation of various types of zeolites, such as ZSM-5 [15,16,17], NaA, and NaY [18] has already been reported. MFI-type zeolites exhibit a unique pore structure composed of intersecting straight and zig-zag channels of 0.5–0.6 nm width [19]. The hydrothermal transformation of RHA into ZSM-5 using TPABr [20,21] or TPAOH [15,17,22] as template in the presence of NaOH has also been reported. In these studies, RHA was first transformed into a sodium silicate solution, which was then used as the silica precursor for zeolite synthesis [17]. In this way, only a unimodal micropore system was obtained. This process has some disadvantages, such as high energy costs and the complexity of the process. Other studies have investigated the influence of heterogeneous phases (impurities) in biogenic silica, i.e., diatomite [14] and RHA [22], on the synthesis of MFI-type zeolites. Recently, we reported the one-step transformation of diatomite into MFI-type zeolite with tetrapropylammonium hydroxide (TPAOH), which combines two functions (the dissolution and reorganization of silica) in one molecule [15]. In the present work, the partial and total transformation of amorphous RHA particles into zeolite was investigated. It will be shown that the choice of the structure-directing agent (SDA) and silica sources drastically influences the pathway of crystallization and the properties of the resulting MFI-type zeolite. In this contribution, we report our systematic studies on the hydrothermal transformation of amorphous RHA into MFI-type zeolites. The role of template has been studied using two different templates, i.e., tetrapropylammonium hydroxide (TPAOH for the direct transformation of RHA into zeolite) and *n*-propylamine (PA for the pseudomorphic transformation of RHA into zeolite). Additionally, MFI-type zeolites have been produced via a two-step pseudomorphic transformation of RHA into MCM-41 and then into zeolite, which has not yet been described in the literature.

## 2. Results and Discussion

**Figure 1:**
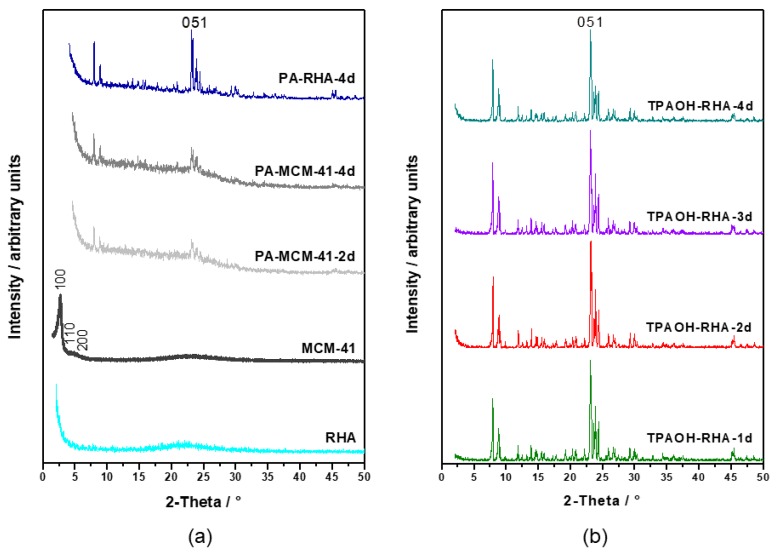
XRD patterns of the initial rice husk ash (RHA), the derived MCM-41 material, and the as-synthesized MFI-type zeolites obtained in procedure 1 from the pseudomorphic transformation of RHA or MCM-41 by using PA template (**a**) and direct transformation of RHA into MFI by using TPAOH template (procedure 2) at different crystallization times (**b**).

Figure 1a,b show the evolution of the XRD patterns of RHA (Figure 1a, bottom), MCM-41 formed via the pseudomorphic transformation of RHA (Figure 1a, penultimate), and MFI-type zeolite phases produced by using PA (Figure 1a, the first three) and TPAOH (Figure 1b) templates at 175 °C as a function of time. The XRD pattern of the RHA (Figure 1a) is characterized by a broad halo in the 2θ range between 17° and 27°, indicating the presence of an amorphous phase. The high intensity in the range below 5° 2θ also indicates typical features associated with an MCM-41-type material with a poor long-range order. This can be explained by small dimensions of ordered domains resulting from a highly disordered mesostructure in the starting material for pseudomorphic transformation [9,23].

After 4 days of hydrothermal treatment with propyl amine, reflections typical for MFI-type zeolite (main characteristic peaks at 2θ = 7.9°, 8.7°, 23.1°) are visible for both starting materials (RHA or MCM-41). The transformation of RHA with TPAOH (Figure 1b) shows MFI reflections with high intensity already after 1 day of hydrothermal treatment, while pseudomorphic transformation with PA as the structure-directing agent showed a much lower long-range order as compared with direct transformation using TPAOH. Table 1 summarizes the dependencies of the product properties, pH value in the filtrate, relative crystallinity, and molar SiO_2_/Al_2_O_3_ ratio on crystallization time. The main diffraction peak at about 2θ = 23.23° was utilized to calculate the relative crystallinity (phase content in the solid) of the obtained MFI-type zeolite samples. Only the transformation product from TPAOH-containing solutions exhibits a high crystallinity after 4 days of hydrothermal treatment (TPAOH-RHA-4 d). This indicates that the RHA was completely transformed into zeolite in this case, while with PA a highest crystallinity of only 42% was obtained after 4 days. The two-step conversion via MCM-41 resulted in even lower crystallinities of only 23% after 4 days of treatment.

The samples collected from the pseudomorphic transformation of RHA or MCM-41 using PA even after a period of 6 h and 1 day exhibit amorphous phases. However, the characteristic reflections corresponding to MFI-type zeolites (2θ = 7.9°, 8.7°, 23.1°) start appearing after 1 day. Furthermore, the relative crystallinity of the synthesis products showed a slow increase up to 4 days with the value of 42% and 23% for the PA-RHA-4 d sample and the PA-MCM-41-4 d sample, respectively.

**Figure 2:**
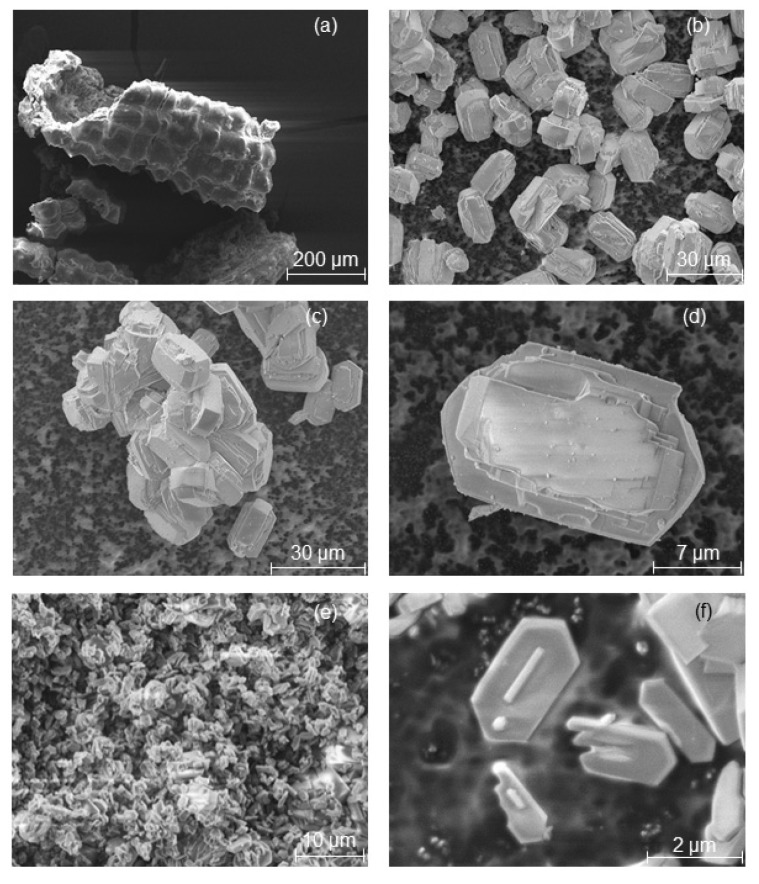
SEM images of the initial RHA (**a**) and the as-synthesized MFI-type zeolite obtained from the direct transformation of RHA using TPAOH for 2 days (**b**–**d**) and 4 days crystallization time (**e**,**f**).

**Figure 3:**
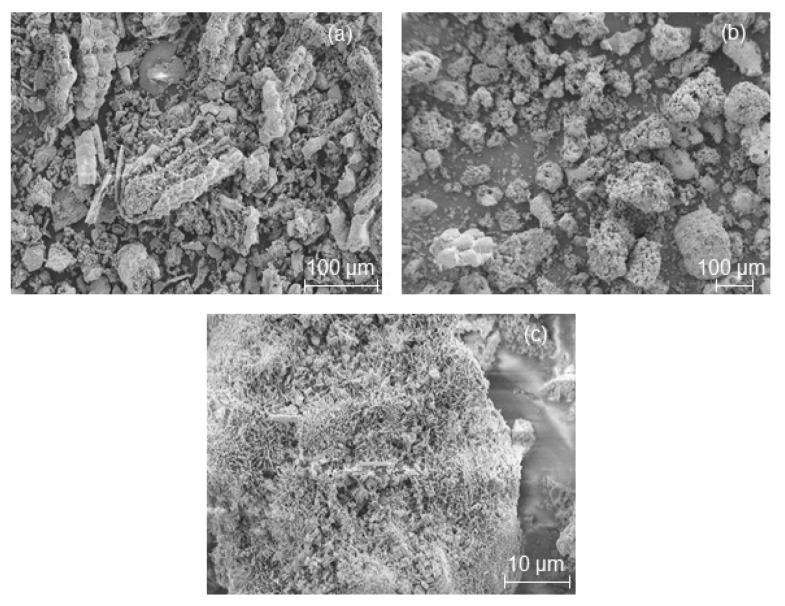
SEM images of MCM-41 formed by the pseudomorphic transformation of RHA (**a**) and the as-synthesized MFI-type zeolite obtained from the pseudomorphic transformation of MCM-41 by using PA after 4 days crystallization time (**b**) and its outer epidermis (**c**).

**Figure 4:**
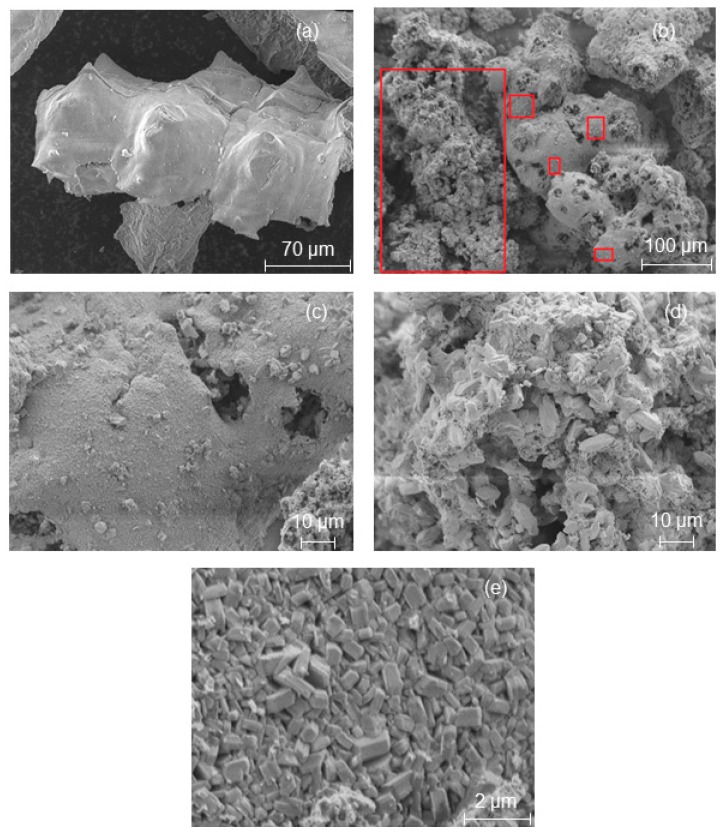
SEM images of the initial RHA (**a**) and the as-synthesized MFI-type zeolite obtained from the pseudomorphic transformation of RHA by using PA after 4 days (**b**) crystallization time, its outer epidermis (**c**–**e**).

In other words, TPAOH seems to cause a faster dissolution of the Si and Al in the starting materials and crystallization in the form of a zeolite framework. This observation clearly demonstrated the influence of template structure on the formation rates of zeolite crystals (see the relative crystallinity values in Table 1) and the resulting crystal shapes (Figure 2, Figure 3 and Figure 4).

Also, the product properties, such as the SiO_2_/Al_2_O_3_ molar ratio and the pH value of the filtrate, depend on the crystallization time (Table 1). The pH value of the filtrate and the yield of the solid decreased with increasing crystallization time.

Figure 2, Figure 3 and Figure 4 show ESEM micrographs of the MFI-type zeolites obtained from the transformation of RHA and MCM-41 samples. The ESEM micrograph of TPAOH-RHA-2 d (Figure 2b) reveals the formation of typical MFI zeolite crystals after 2 days of hydrothermal treatment (with a relative crystallinity of 78%). As the crystallization time increases, the relative crystallinity increases for TPAOH-RHA-4 d (Table 1). Figure 2d shows uniform crystals in the whole sample after the direct transformation of the RHA. An increase in the hydrothermal treatment time to 4 days for RHA results in a reduction of the crystal size from ~20 µm to around 2 to 3 µm (Figure 2e,f).

In order to show that the shape of the starting RHA or MCM-41 material based on RHA remains intact during their pseudomorphic transformation (in the presence of propyl amine) into MFI-type zeolites, scanning electron micrographs were taken (Figure 3 and Figure 4). The characteristic macroscopic morphology (double rods or a lamellar and backbone structure) of RHA (Figure 2a and Figure 4a) was completely preserved after a partial pseudomorphic transformation of the MCM-41 (Figure 3a) or RHA phase into MFI-type zeolites (Figure 3b and Figure 4b (red spots)) [9]. This effect is observed for the first time in this study. In addition, a high magnification SEM image (Figure 3c and Figure 4c) reveals a rough surface texture of the obtained RHA particles after pseudomorphic transformation. Hence, the nanostructured zeolite formed on the outer surface of RHA either needle-like crystals (Figure 3c) or small zeolite crystals in a size between 7 and 9 µm (Figure 3c and Figure 4c–e) by Ostwald ripening. This is probably due to the presence of larger ordered domains of nanostructured zeolite at the outer surface of the RHA rather than smaller domains in their centers, whereas rounded prisms were formed in the case of direct transformation in the presence of TPAOH (Figure 2d).

The above results show that two different structure-directing agents can dramatically influence the morphology of the resulting MFI-type zeolite as well as the preservation of the RHA’s morphology.

**Figure 5:**
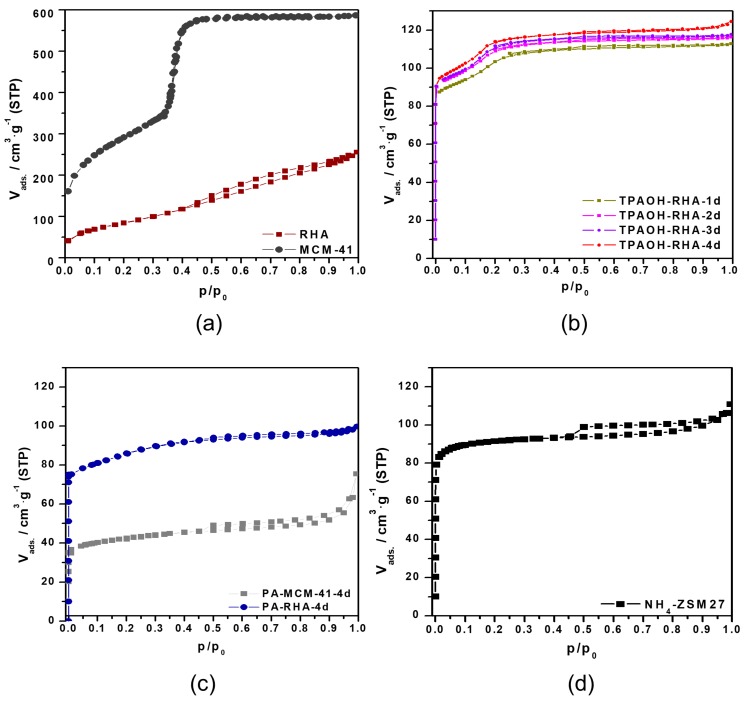
Nitrogen sorption isotherms of RHA and MCM-41 (**a**), the MFI-type zeolite obtained from the direct transformation of RHA using TPAOH (**b**) and pseudomorphic transformation of RHA or MCM-41 by using PA (**c**), and of the industrial MFI-type reference zeolite (NH_4_-ZSM27) sample (**d**) [15].

Figure 5a–c show the nitrogen physisorption isotherms for initial RHA, MCM-41, and MFI-type zeolites samples after different hydrothermal treatment times. The industrial MFI-type reference zeolite (NH_4_-ZSM27) (Figure 5d) shows a nitrogen isotherm of type I [24], which is due to the sole presence of micropores [15].

The nitrogen sorption isotherm of RHA and ordered mesoporous MCM-41 prepared by pseudomorphic transformation exhibits a type IV isotherm. However, only a slight and less steep hysteresis occurred between the adsorption and desorption branch at about p/p_0_ 0.45 to 1.00 of the RHA isotherm. This is due to the presence of less but larger meso- and macropores in the particles of RHA. After transformation into MCM-41, a sharp step at about p/p_0_ = 0.37 of its isotherm is obtained. Thus, the synthesis of MCM-41 by the pseudomorphic transformation of RHA is a good method to obtain MCM-41 material with a high specific surface area (A_BET_ = 1210 m^2^·g^−1^), a high specific mesopore volume (V_meso_ = 1.0 cm^3^·g^−1^) [6,9,25], and the morphological characteristics of RHA shapes.

In accordance with MFI-type zeolites obtained from other biomass materials, e.g., diatomite [15], all zeolite samples synthesized in this study exhibit type I isotherms with an additional uptake step for nitrogen in the p/p_0_ region of 0–0.25. The isotherms show a horizontal plateau at p/p_0_ between 0.25 and 1 as a further feature. The variation in the aluminum content in the zeolite samples (see Table 2) affects the shape of the individual isotherms. This is clearly manifested in the low relative pressure region between p/p_0_ 0.1 and 0.25. The isotherms of the sample series exhibit two steps which have been deeply explained in our previous study, but it is more clear in the samples formed by the direct transformation of RHA using TPAOH (Figure 5b) [15]. The slope of these steps decreases with increasing crystallization time and might be due to the increasing aluminum content in the zeolite framework. The first step is characterized by a sharp knee at p/p_0_ lower than 0.1 and can be interpreted with the occupation of an increasing number of sites in the channels and intersections of MFI-type zeolites with nitrogen molecules. The second step in the isotherms of the MFI-type zeolites based on RHA characterizes an additional nitrogen adsorption at a p/p_0_ region between 0.12 and 0.25 (Figure 5b,c). This step includes the interaction between the adsorbates, followed by a reorientation of the molecules to allow for the most favorable quadrupole–quadrupole interactions. As a result, an increased ordering of the nitrogen molecules under the formation of a chain-like structure was observed [26]. The two steps are absent in the nitrogen physisorption isotherm of the reference material NH_4_-ZSM27 with a lower SiO_2_/Al_2_O_3_ molar ratio (Figure 5d). The specific micro-, meso- and total pore volumes, micropore and external surface area values of the MFI-type zeolite samples were calculated on the basis of the t-plot method and are summarized in Table 2. Although the t-plot was done in the layer thickness range between 0.35 and 0.5 nm, which relates to the adsorption points below 0.25 p/p_0_, the calculated textural values are relatively lower for those isotherms which exhibit the additional nitrogen uptake step between 0.1 and 0.25 p/p_0_ than the values for the reference zeolite. For example, for sample TPAOH-RHA-4 d the XRD and nitrogen adsorption isotherm show similar characteristics as the reference zeolite (100% crystallinity and nitrogen uptake close to 90 cm^3^/g nitrogen gas STP in Figure 5b), but the calculated micropore volume of TPAOH-RHA-4 d is only 0.09 cm^3^/g instead of 0.14 cm^3^/g according to the t-plot calculation, obviously because of the different shape of the adsorption isotherms in the p/p0 range between 0.1 and 0.25. Due to this discrepancy the textural data given in Table 2 could only be used to analyse relative but not quantitative tendencies of transformation.

The amount of adsorbed nitrogen increases for MFI-type zeolite samples by rising the crystallization time (Figure 5b,c). The materials are characterized by a specific micropore surface area of 205 m^2^·g^−1^ for TPAOH-RHA-4 d, 87 m^2^·g^−1^ for PA-RHA-4 d and 54 m^2^·g^−1^ for PA-MCM-41-4 d. Their micropore volumes amount to 0.09 cm^3^·g^−1^, 0.06 cm^3^·g^−1^ and 0.04 cm^3^·g^−1^, respectively (Table 2), which indicates that 4 days transformation of RHA in the presence of TPAOH results in a higher zeolite (micropore) content than transformation in the presence of PA. This is in agreement with the XRD derived crystallinity values given in Table 1. Furthermore, a slight hysteresis occurred between the adsorption and desorption branch of the isotherms. The lower closure point of the hysteresis loops at about p/p_0_ = 0.45 was almost the same for all MFI-type zeolite samples. This is due to pore blocking effects. Some large pores are connected with the external surface of crystallites or particles via smaller micropores. A similar behavior is observed for the reference material (Figure 5d) [15].

The textural properties of the transformed materials are clearly influenced by the SiO_2_/Al_2_O_3_ molar ratios in the starting system and the resulting MFI-type zeolite samples [15]. In the case of SiO_2_/Al_2_O_3_ molar ratios >100 (Table 1 and Table 3) excessive and unselective dissolution of the silica network of the zeolites is observed, which leads to the creation of relatively large external surface areas up to 270 m^2^/g and high mesopore volumes up to 0.29 cm^3^/g for the RHA sample transformed in PA containing synthesis mixture via the MCM-41 intermediate (PA-MCM41-4 d), which in turn exhibits almost 50% less micropore volume compared to the TPAOH transformation products.

Additionally, the textural characterization of the transformation products shows that the transformation of RHA or MCM-41 into MFI-type zeolites caused the introduction of microporosity and reduction of the original mesoporosity. The effect is also shown in the isotherms (p/p_0_ region > 0.3) of these samples (Figure 5c). As the result, the external surface area decreased as compared to the initial RHA or MCM-41 (Table 2 and Table 3). These observations together with the SEM images in Figure 3 and Figure 4 are indications for a “real” at least partial pseudomorphic transformation of RHA or RHA-based MCM-41 starting materials into MFI-type zeolites.

## 3. Materials and Methods

### 3.1. Starting Materials

The precursor RHA was obtained by a specific thermo-chemical treatment of RH supplied from the Kafr El-Daowar locality in Egypt (E-RH, Kafr El-Daowar, Egypt). The process included thoroughly washing the husk sample with water, dry milling, and leaching with citric acid. The leaching process was applied in two stages: for 180 min at 50 °C and then at 80 °C for 60 min. After washing and drying, the leached sample was subjected to a heat treatment in a muffle furnace for four sequential steps: 310 °C for 60 min, 400 °C for 120 min, 510 °C for 300 min, and 600 °C for 30 min [1].

Ordered mesoporous MCM-41 was prepared via pseudomorphic transformation of the purified RHA. The molar composition of the final reaction mixture was 1 SiO_2_ (RHA): 0.20 C_16_H_33_(CH_3_)_3_N: 0.20 OH^−^: 140 H_2_O and pH = 12.2 of 0.08 M CTAOH. One gram of RHA was poured into 42 mL of the surfactant solution. The reaction mixture was stirred (~500 rpm, RCTB, IKA, Staufen im Breisgau, Germany) for 1 h at room temperature. The hydrothermal synthesis was performed in a PTFE-lined stainless steel autoclave (60 mL, type 4744, Parr Instrument GmbH, Frankfurt am Main, Germany). After filling the autoclave with the reaction mixture, the system was degassed at 0.8 mbar in a vacuum cabinet for 15 min. Thereafter, the autoclave was sealed and heated at 120 °C for 6 days to form the surfactant-silica mesophase. The autoclave was removed from the oven and quenched in cold water. The obtained product was filtered, washed with deionized water, and dried at 110 °C overnight. To remove the surfactant, the dried sample was calcined in static air employing a muffle furnace (type N 11/H, Nabertherm, Lilienthal, Germany) at 550 °C for 5 h with a linear temperature ramp of 10 °C min^−1^ and two plateaus of 120 min at 200 and 400 °C [9].

The composition and textural properties of the prepared RHA and MCM-41 samples are listed in Table 3. The initial RHA and MCM-41 materials were used as the sole silica and alumina sources for the zeolite synthesis.

RHA and MCM-41 were characterized by a SiO_2_/Al_2_O_3_ molar ratio equal to 413 and 1119, respectively. However, the characteristics of RHA vary depending on the treatment methods, RH sources, geographical location (such as cultivation area, soil fertility, weather, and irrigation factor), year of harvest, treatment method of RH, and degree of grinding [27,28]. They also depend upon the preservation of cellular structure and the extent of amorphous material within the structures [29].

Tetrapropylammonium hydroxide solution (1.0 M TPAOH in H_2_O) and monopropylamine ≥99% have been provided by Sigma-Aldrich, Steinheim, Germany. A fine-grained commercial (industrial) MFI-type zeolite in the ammonium form (abbreviated as NH_4_-ZSM27) with a molar SiO_2_/Al_2_O_3_ ratio of 54 was used as reference material (Clariant, Frankfurt am Main, Germany). Solutions of 0.08 M hexadecyltrimethylammonium hydroxide (CTAOH) were prepared by a double ion exchange of hexadecyltrimethylammonium bromide (CTABr, purum, ≥96.0%, Fluka, Germany), using an AMBERSEP^®^ 900 OH ion exchange resin (total exchange capacity 0.8 meq mL^−1^, (OH^−^) form, Alfa Aesar, Karlsruhe, Germany).

### 3.2. Synthesis of MFI-Type Zeolites

The RHA and MCM-41 precursors were applied as simultaneous silica and alumina sources in two separate procedures.

In both synthesis procedures, the hydrothermal treatment was performed in a stainless steel autoclave lined with PTFE (volume 45 cm^3^, Nr. 4744, Parr Instrument GmbH, Frankfurt am Main, Germany) without stirring and at 175 °C (oven type U50, Memmert, Büchenbach, Germany) for a desired period of time at autogeneous pressure. After each synthesis run, the autoclave was removed from the oven and quenched in cold water. The obtained product was filtered; the filter residue was washed with deionized water until pH 8 was reached, dried at 100 °C overnight, and finally calcined in air at 550 °C for 5 h using a muffle furnace (type N 11/H, Nabertherm, Lilienthal, Germany) with a heating rate of 1 °C min^−1^.

#### 3.2.1. Procedure 1

In a typical procedure, the pseudomorphic transformation of RHA into MFI-type zeolite was realized by adding either E-RHA or derived MCM-41 (based on E-RHA) into the monopropylamine (PA)-containing alkaline NaOH solution without stirring. The composition of the reaction mixture, expressed in mole ratios of the oxides, was as follows:

E-RHA:
1 E-RHA: 0.00242 Al_2_O_3_: 15.12 PA: 0.2 Na_2_O: 19 H_2_O with an initial pH value of 11.9.
MCM-41:
1 MCM-41: 0.000894 Al_2_O_3_: 15.12 PA: 0.2 Na_2_O: 19 H_2_O with an initial pH value of 12.0.
In both cases, the reaction mixture was prepared with the following mass composition:
1.0 g E-RHA or MCM-41, 15.0 g PA, 0.1 g NaOH and 3.9 g H_2_O.

#### 3.2.2. Procedure 2

In the second procedure, tetrapropylammonium hydroxide solution (TPAOH) was used for a direct transformation of RHA into MFI-type zeolite. The molar composition of the reaction system was:

- 1 E-RHA: 0.00242 Al_2_O_3_: 0.135 TPA_2_O: 0.27 OH^−^: 57 H_2_O with an initial pH value of 12.8 for the direct transformation of RHA into zeolite. Accordingly, the initial mass composition of the reaction mixture was: 1.0 g E-RHA, 4.44 g 1.0 M TPAOH and 13.73 g H_2_O.

In procedure 2, the initial reaction mixture was stirred (500 rpm; RCTB, IKA, Staufen im Breisgau, Germany) for 1 h at room temperature.

### 3.3. Methods

X-ray powder diffraction (XRD) patterns were recorded with an XRD 7 diffractometer (GE Seifert, Ahrensburg, Germany) with Cu-Kα radiation at a 40 kV acceleration voltage and a 30 mA beam current. The reflection was scanned from 1 to 50° 2θ in the Bragg–Brentano geometry, with a step width of 0.05° and a step time of 1 min.

The relative crystallinity (%) in the reaction products was obtained from the integrated intensity of the main X-ray reflection (2θ ~ 23.1°) of the as-synthesized MFI-type zeolite in relation to the integrated intensity of the main X-ray reflection (2θ ~ 23.1°) of an industrial MFI reference zeolite [15].

The SiO_2_/Al_2_O_3_ molar ratio and the concentration of the impurities in the as-synthesized zeolite were calculated on the data determined by inductively coupled plasma optical emission spectrometry (ICP-OES, Spectro Ciros CCD, Spectro Analytical Instruments GmbH, Kleve, Germany). The standard relative deviations were between ±3% and ±5%. Prior to the ICP-OES analysis, the samples were treated as follows: 0.1 g of solid sample was weighed and digested together with 8 mL of a mixture of 40% HF, 2 mL 65% HNO_3_, and 2 mL 32% HCl in a PTFE container inside a microwave oven (800 W, 40 min). The sample was cooled and diluted to 250 mL with bi-distilled water.

The pore structure of the samples was analyzed by nitrogen adsorption (ASAP 2010, Micromeritics, DR-Norcross, GA, USA). Before the analysis, all samples were degassed at 200 °C under vacuum for 8 h. The specific micropore volume, the specific micropore area, and the external surface area of MFI-type zeolites were estimated by the t-plot method supposing a layer thickness between 0.35 and 0.5 nm. The surface morphology of the materials was studied by using an Environmental Scanning Electron Microscope (ESEM) (Philips ESEM XL 30 FEG, Amsterdam, The Netherlands) equipped with an energy dispersive X-ray spectrometer (EDX/EDAX). The alkalinity of the filtrate after synthesis was measured with a pH meter (pH 330i, WTW, Weilheim, Germany) with an accuracy of 0.05.

## 4. Conclusions

In this study, the influence of the following parameters on the transformation process of biogenic silica into MFI-type zeolites was investigated:

- Different pretreated starting materials: either rice husk ash (RHA) or MCM-41 prepared via the pseudomorphic transformation of RHA;
- Two structure-directing agents (SDA): tetrapropylammonium hydroxide (TPAOH) for the direct transformation of RHA into zeolite or n-propylamine (PA) for the pseudomorphic transformation of either RHA or RHA-derived MCM-41 into zeolite.

The RHA and MCM-41 precursors served as simultaneous silica and alumina sources. The choice of the SDA and silica sources influenced the pathway of the transformation process and the properties of the resulting MFI-type zeolites.

MFI-type zeolites obtained from RHA-derived MCM-41 displayed a lower relative crystallinity (23%) and microporosity (specific micropore area = 54 m^2^·g^−1^, specific micropore volume = 0.04 cm^3^·g^−1^) than those obtained from RHA (relative crystallinity of 42%, specific micropore area = 87 m^2^·g^−1^, and specific micropore volume = 0.06 cm^3^·g^−1^) after 4 days of transformation. This indicates differences in the pseudomorphic transformation of the two different starting materials.

In the case of RHA and TPAOH, it was found that TPAOH accelerated the RHA dissolution and crystallization uniformly in the whole volume. This resulted in achieving the maximum value of the relative crystallinity after 4 days, which is due to a total dissolution of amorphous RHA before the crystallization of prismatic crystals. The transformation was not pseudomorphic, as the macroscopic shape was not maintained.

When using PA, for both RHA and RHA derived MCM-41, needle-like crystals were formed on the outer surface of the particles. The macroscopic shape of the particles was preserved during the two different transformation procedures. However, the two-step procedure (transformation of RHA based MCM-41) finally resulted in zeolite-based materials with better textural properties (total pore volume and surface area) as compared to the one-step procedure using RHA. These effects were observed for the first time.

The obtained results are opening up a sustainable way to produce tailor-made zeolite-based materials from biogenic silica for applications in catalysis, adsorption, and sensor technology.

## Figures and Tables

**Table 1:** Results of the hydrothermal synthesis of MFI-type zeolites obtained from the pseudomorphic transformation of RHA and MCM-41 by using PA template and direct transformation of RHA using TPAOH template after crystallization times between 6 h and 4 days.

Zeolite Name	pH of Filtrate	Powder Product/wt. %	Relative Crystallinity/%	SiO_2_/wt. %	Al_2_O_3_/wt. %	Molar Ratio SiO_2_/Al_2_O_3_ ***
TPAOH-RHA-6 h	12.08	99.5	22	--- *	--- *	--- *
TPAOH-RHA-12 h	11.92	99.7	51	--- *	--- *	--- *
TPAOH-RHA-1 d	11.85	93.5	63	81.2	0.24	574
TPAOH-RHA-2 d	11.75	88.0	78	86.7	0.30	485
TPAOH-RHA-3 d	11.52	81.7	93	94.0	0.37	440
TPAOH-RHA-4 d	11.38	72.7	100	97.3	0.41	405
PA-RHA-6 h	11.64	99.0	0	--- *	--- *	--- *
PA-RHA-1 d	11.55	97.4	0	--- *	--- *	--- *
PA-RHA-2 d	11.23	95.2	11	75.8	0.04 **	3620
PA-RHA-4 d	11.11	92.1	42	87.3	0.05 **	2863
PA-MCM-41-6 h	11.78	98.9	0	--- *	--- *	--- *
PA-MCM-41-1 d	11.45	97.3	0	--- *	--- *	--- *
PA-MCM-41-2 d	11.37	95.8	7	77.3	0.04 **	3290
PA-MCM-41-4 d	11.17	93.1	23	81.1	0.05 **	2759
MFI-type zeolite reference *	--- *	--- *	100	--- *	--- *	54

* Not determined; ** rounded; *** The SiO_2_/Al_2_O_3_ molar ratio was based on ICP-OES data (see experimental).

**Table 2:** Textural properties of MFI-type zeolites obtained from the direct transformation of RHA using TPAOH and pseudomorphic transformation of RHA or MCM-41 by using PA.

Sample Name	Specific Micropore Area/m^2^·g^−1 1^	External Surface Area/m^2^·g^−1 1^	Specific Micropore Volume/cm³·g^−1 1^	Specific Mesopore Volume/cm³·g^−1 2^	Total Pore Volume/cm³·g^−1 3^
TPAOH-RHA-1 d	119	113	0.05	0.06	0.11
TPAOH-RHA-2 d	148	125	0.06	0.08	0.14
TPAOH-RHA-3 d	172	136	0.08	0.09	0.17
TPAOH-RHA-4 d	205	154	0.09	0.10	0.19
PA-RHA-4 d	87	112	0.06	0.17	0.23
PA-MCM-41-4 d	54	270	0.04	0.29	0.33
* NH_4_-ZSM27	270	43	0.14	0.03	0.17

^1^ Specific micropore surface area, external surface area, and specific micropore volume are calculated from the t-plot at a layer thickness range between 0.35 and 0.5 nm; ^2^ specific mesopore volume is the difference between total pore volume and micropore volume; ^3^ total pore volume is calculated at p/p_0_ = 0.995; * industrial MFI-type reference zeolite.

**Table 3:** Chemical compositions, quantitative phase analysis, pore characteristics, and particle size analysis of the RHA and MCM-41 samples [1,9,22].

Properties of Modified Rice Husk Ash Samples		RHA	MCM-41
Chemical composition/wt. %	SiO_2_	97.70	98.00
	Al_2_O_3_	0.25	0.10
	Others *	2.05	1.90
Molar ratio SiO_2_/Al_2_O_3_		413	1119
Quantitative XRD phase analysis/wt. %		100% amorphous phase	completely transformed (100%)
Specific surface area/m^2^·g^−1^		313	1210
Mesopore volume/cm^3^·g^−1^		0.38	1.00
Pore width distribution (Av. pore diameter)		very broad (5.0 nm)	uniform (4.1 nm)
Av. particle size dp/µm **		23	198

* Other inorganic oxides; ** Av. particle size for the sum of smallest 10%, 50%, and 90% of the analyzed powder of RHA and MCM-41.
